# Cloning and characterization of microRNAs from rainbow trout (*Oncorhynchus mykiss*): Their expression during early embryonic development

**DOI:** 10.1186/1471-213X-8-41

**Published:** 2008-04-15

**Authors:** Raghuveer K Ramachandra, Mohamed Salem, Scott Gahr, Caird E Rexroad, Jianbo Yao

**Affiliations:** 1 Laboratory of Animal Biotechnology and Genomics, Division of Animal and Nutritional Sciences, West Virginia University, Morgantown, WV 26506, USA; 2 U.S. Department of Agriculture, Agricultural Research Service, National Center for Cool and Cold Water Aquaculture, Kearneysville, WV 25430, USA

## Abstract

**Background:**

Current literature and our previous results on expression patterns of oocyte-specific genes and transcription factors suggest a global but highly regulated maternal mRNA degradation at the time of embryonic genome activation (EGA). MicroRNAs (miRNAs) are small, non-coding regulatory RNAs (19–23 nucleotides) that regulate gene expression by guiding target mRNA cleavage or translational inhibition. These regulatory RNAs are potentially involved in the degradation of maternally inherited mRNAs during early embryogenesis.

**Results:**

To identify miRNAs that might be important for early embryogenesis in rainbow trout, we constructed a miRNA library from a pool of unfertilized eggs and early stage embryos. Sequence analysis of random clones from the library identified 14 miRNAs, 4 of which are novel to rainbow trout. Real-time PCR was used to measure the expression of all cloned miRNAs during embryonic development. Four distinct expression patterns were observed and some miRNAs showed up-regulated expression during EGA. Analysis of tissue distribution of these miRNAs showed that some are present ubiquitously, while others are differentially expressed among different tissues. We also analyzed the expression patterns of Dicer, the enzyme required for the processing of miRNAs and Stat3, a transcription factor involved in activating the transcription of miR-21. Dicer is abundantly expressed during EGA and Stat3 is up-regulated before the onset of EGA.

**Conclusion:**

This study led to the discovery of 14 rainbow trout miRNAs. Our data support the notion that Dicer processes miRNAs and Stat3 induces expression of miR-21 and possibly other miRNAs during EGA. These miRNAs in turn guide maternal mRNAs for degradation, which is required for normal embryonic development.

## Background

Embryos are dependent on maternally stored mRNAs for their transcriptional needs until their own transcription machinery is functional. The initiation of embryonic gene expression, embryonic genome activation (EGA), varies greatly with species. While it occurs by the 2-cell stage in mice, it does not take place until mid-blastula in teleosts [1] and amphibians [2]. Three reasons have been suggested to explain why the embryonic genome is not able to transcribe mRNAs before EGA; namely (i) epigenetic and chromatin mediated repression, (ii) insufficient transcription machinery and (iii) lack of sufficient time for the chromosome to transcribe while it is undergoing rapid cell divisions [3]. In zebrafish, the maternal-zygotic transition is characterized by asynchronous cell division, lengthening of cell cycles, cell motility and initiation of zygotic transcriptional machinery [1]. After this activation, the embryo becomes increasingly dependent on its own transcription and exhausts the maternally stored mRNAs. It was recently shown that it is critical to degrade maternally inherited mRNAs to achieve normal morphogenesis which is delayed if these inherited mRNAs are not degraded [4]. This suggests a tightly controlled regulatory mechanism in nature to degrade maternal mRNAs at the time of EGA.

Previously, we showed that the transcripts for several transcription factors [5] and a novel oocyte-specific protein [6] are degraded during/around the time of EGA in rainbow trout. Apart from the involvement of TATA binding protein (TBP) in regulating maternal mRNA degradation [7], little information is available on how maternal mRNA degradation is regulated. Mechanisms by which this degradation is accomplished escaped scientific attention until recently. It was hypothesized by Schier [3] that microRNAs (miRNAs) are involved in these degradation processes.

MicroRNAs are small, 19–23 nucleotides (nt) non-coding RNAs that bind to recognition sequences on 3'-untranslated regions (3'-UTRs) of mRNAs and target them for degradation in cases of high complementarity or translational repression in cases of partial complementarity. Interestingly, a miRNA can bind to a specific recognition sequence on 3'-UTR of up to 200 transcripts and each mRNA could have recognition sequences for many miRNAs [8]. The current miRNA model is well suited to explain maternal mRNA degradation because miRNAs degrade mRNAs in a specific and large scale manner which is the case during EGA. Absence of all miRNAs caused by the deficiency of Dicer, an enzyme that is required for processing miRNAs, results in severe early embryonic deformities and faulty brain morphogenesis [9]. In addition, Girldez and coworkers found that miR-430 leads to rapid deadenylation and degradation of maternal mRNAs [4].

To identify miRNAs that might be important for early embryogenesis in rainbow trout, we constructed a miRNA library from a pool of unfertilized eggs and early stage embryos. We report here the identification of 14 rainbow trout miRNAs and their expression profiles during early embryonic development. Our analysis showed that all 14 miRNAs are present in the early stage embryos examined with multiple expression patterns. In addition, we present data to show that Dicer, the enzyme required for the processing of miRNAs, is abundantly expressed during EGA, and Stat3 which activates transcription of miRNA-21, is up-regulated before the onset of EGA, supporting the important roles of these proteins in activation/processing of miRNAs which in turn degrade maternal mRNAs during early embryonic development.

## Results and Discussion

### Cloning and identification of rainbow trout microRNAs

**Figure 1:**
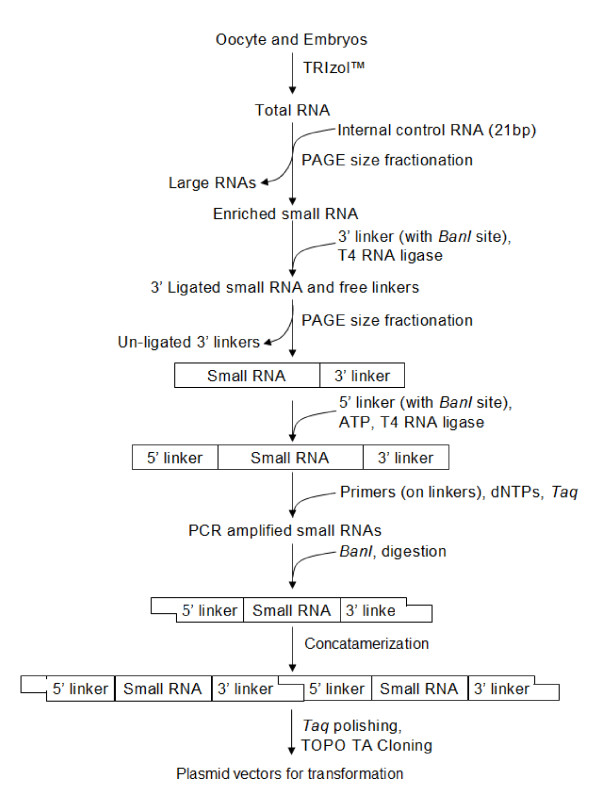
**Schematic diagram showing method used to clone rainbow trout miRNAs. **MirCat™ (IDT DNA Technologies, Coralville IA) miRNA cloning kit was used with minor modifications.

**Figure 2:**
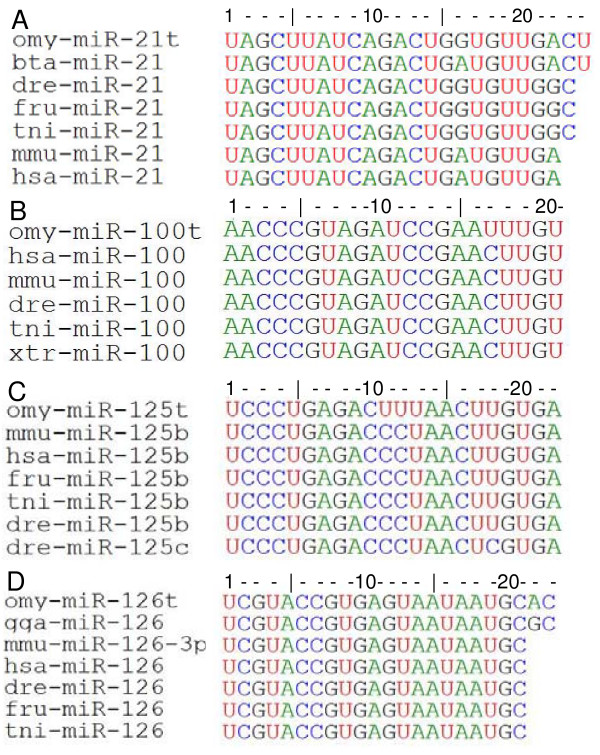
Alignment of the novel rainbow trout miRNAs with highly homologous miRNAs from other species using BioEdit program.

A schematic diagram of the method used to construct the miRNA library is depicted in Fig. 1. The library was constructed using small RNAs isolated from a pool of oocytes and early embryos. Random clones were picked and screened for presence of inserts by colony PCR before sequencing. A total of 150 clones were sequenced. Sequence analysis identified 32 small RNAs that show significant similarities with published miRNAs (Sanger database Version 10.0 [10]). Names of the rainbow trout miRNA were assigned based on the homologies between the cloned sequence and published miRNA sequences at Sanger database (Table 1) using internationally accepted uniform nomenclature [11]. After excluding redundancy, fourteen unique miRNAs were identified from rainbow trout that are conserved across several species (Table 1). Four miRNAs were novel to rainbow trout characterized as having high homologies with published miRNAs but differed by at least 1 nucleotide. Since we obtained multiple copies of the same sequence in the library, it is unlikely that these are sequencing errors. We annotated the novel miRNAs as omy-miR-100t, omy-miR-21t, omy-miR-125t and omy-miR-126t (names end with 't' for trout, Table 1 and Fig. 2). These miRNAs only observed in the rainbow trout (omy-miR-100t, omy-miR-21t, omy-miR-125t and omy-miR-126t) are of special interest because of their unique sequences and possibly unique targeting mechanisms. These miRNAs differ by at least one nucleotide and this difference might have profound impact on target recognition and post-transcriptional regulation. Omy-miR-21t has a G to A mismatch with miR-21 of cows at position 16 but this mismatch seems to be common for all fishes (miR-21 of zebrafish, fugu and pufferfish, Fig. 2A). MiR-21 from all these three fishes is 23 nucleotides long whereas in humans and mice, miR-21 is only 22 bases long. However, A to G mismatch between trout and other fishes at position 22 is absent between trout and mammals (Fig. 2A). In case of omy-miR-100t, there is a U to C single base substitution at position 17 and this seems to be specific to rainbow trout (Fig 2B). So is the case with omy-miR-125t, where the mismatch is a C to U at positions 11 and 12 (Fig 2C). The presence of omy-miR-125b (identical to miR-125b of other species) and omy-miR-125t, specific to rainbow trout, suggests another layer of complexity in post-transcriptional regulation by miRNAs. omy-miR-126t sequence is identical to that of chicken, mammals and fishes up to position 20, but has an A to G mismatch at position 21 as compared to chicken miR-126 (Fig 2D). These mismatches may not have profound influences on target recognition and mRNA degradation because they are not in the seed sequence (first 6–8 nucleotides from 5' end) but they are likely fine-tune the expression of their targets [12].

**Table 1:** Cloned rainbow trout miRNAs and primers used for real time PCR analysis.

**MicroRNA**	**Sequence**	**Size**	**Real Time Primer Sequence**	**Conserved in other animals**
omy-miR-21	UAGCUUAUCAGACUGGUGUUGGC	23	TAGCTTATCAGACTGGTGTTGGC	dre, fru, tni
omy-miR-21t	UAGCUUAUCAGACUGGUGUUGAC	23	TAGCTTATCAGACTGGTGTTGAC	see Figure 2A
omy-miR-23a	AUCACAUUGCCAGGGAUUUCCA	22	ATCACATTGCCAGGGATTTCCA	dre, fru, tni, bta
omy-miR-26a	UUCAAGUAAUCCAGGAUAGGCU	22	TTCAAGTAATCCAGGATAGGCT	dre, ssc, ptr, ggo, lla, mml, ppa, fru, tni, bta
omy-miR-30d	UGUAAACAUCCCCGACUGGAAG	22	TGTAAACATCCCCGACTGGAAG	bta, hsa, mmu, rno, gga dre, ptr, ggo, mne, ppa, fru, tni, xtr
omy-miR-92a	UAUUGCACUUGUCCCGGCCUGU	22	TATTGCACTTGTCCCGGCCTGT	dre, ggo, lme, age, ppa, ppy, ptr, mml, sla, lla, mne, fru, tni, bta
omy-miR-100t	AACCCGUAGAUCCGAAUUUGU	21	AACCCGTAGATCCGAATTTGT	see Figure 2B
omy-miR-125a	UCCCUGAGACCCUUAACCUGUG	22	TCCCTGAGACCCTTAACCTGTG	dre, fru, tni, xtr
omy-miR-125b	UCCCUGAGACCCUAACUUGUGA	22	TCCCTGAGACCCTAACTTGTGA	dre, dme, hsa, rno, gga, dps, aga, dre, ssc, ggo, ppa, ssc, age, ppy, ptr, mml, sla, lla,, mne, lca, fru, tni, bta, xtr, mdo
omy-miR-125t	UCCCUGAGACUUUAACUUGUGA	22	TCCCTGAGACTTTAACTTGTGA	see Figure 2C
omy-miR-126t	UCGUACCGUGAGUAAUAAUGCAC	23	TCGTACCGTGAGTAATAATGCAC	see Figure 2D
omy-miR-126*	CAUUAUUACUUUUGGUACGCG	21	CATTATTACTTTTGGTACGCG	mmu, hsa, rno, gga, dre, xtr
omy-miR-200b	UAAUACUGCCUGGUAAUGAUGAU	23	TAATACTGCCTGGTAATGATGAT	gga, xtr
omy-miR-455	UAUGUGCCCUUGGACUACAUCG	22	TATGTGCCCTTGGACTACATCG	dre, fru, tni, gga, xtr

### Expression patterns of miRNAs during early embryonic development

**Figure 3:**
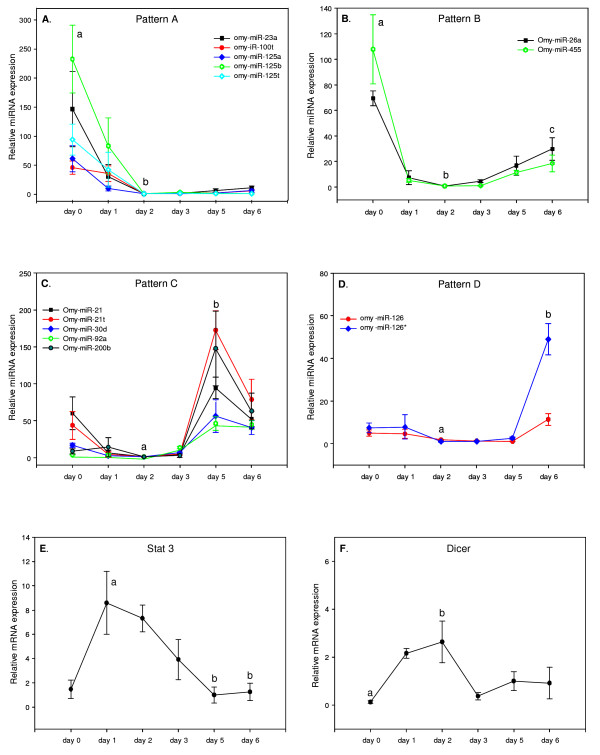
**Expression patterns of rainbow trout miRNAs (A to D), Stat3 (E) and Dicer (F) during early embryogenesis as revealed by real-time PCR analysis.** Embryonic stages analyzed include unfertilized eggs (0-dpf), embryos at 1-dpf, 2-dpf, 3-dpf, 5-dpf and 6-dpf. Quantity of each miRNA or mRNA was normalized to Histone H2A. The means of the normalized gene expression values for each embryonic stage were calculated and expressed as relative fold changes (n = 3, mean ± SEM). Different letters indicate significant difference at P < 0.05.

We analyzed the expression patterns of 14 miRNAs over the first 6 days of rainbow trout development (Fig. 3). The overall pattern of all 14 miRNAs shows a minimum expression in embryos at 2 days post fertilization (dpf). Based on miRNA abundance in embryos before and after 2-dpf, the miRNAs were clustered into 4 distinct patterns. Pattern A, observed for omy-miR-23a, omy-miR-100t, omy-miR-125a, omy-miR-125b and omy-miR-125t, starts with a relatively high expression in unfertilized egg (0-dpf), declines gradually, reaching a very low level in 2-dpf embryos (*P *< 0.05) and remains low levels thereafter (Fig. 3A). Pattern B, which includes omy-miR-26a and omy-miR-455, is similar to pattern A before 2-dpf. After 2-dpf, the expression of these miRNAs increases significantly (*P *< 0.05, Fig. 3B). MicroRNAs in pattern C include omy-miR-21, omy-miR-21t, omy-miR-30d, omy-miR-92a and omy-miR-200b. These miRNAs show a characteristic peak expression at 5-dpf (*P *< 0.05, Fig. 3C). Pattern D miRNAs include omy-miR-126 and omy-miR-126*. These miRNAs show a very significant increase in expression at 6-dpf compared to other stages (*P *< 0.05, Fig. 3D).

The general trend of declined expression of miRNAs from 0-dpf to 2-dpf, especially seen in patterns A and B, is similar to that of maternal transcripts. The increased expression of pattern B miRNAs (between 2-dpf and 6-dpf) probably denotes the onset of EGA (Fig. 3B). The lower expression of all miRNAs in 2-dpf embryos may indicate the transition point from the maternal to the embryonic genomes. Mao et al., determined that onset of the rainbow trout embryonic nuclear activity, genome activation, occurs at the stage 9 which is equivalent to 4-dpf for rainbow trout embryos reared at 8.5°C [13]. In our study, the embryos were incubated at ~13°C which explains the earlier onset of EGA.

Significant increases of miRNA abundance at specific embryonic stages shown in patterns C (5-dpf) and D (6-dpf) suggest important roles for these miRNAs in rainbow trout development; possibly involved in degrading residual maternal mRNAs that remains after EGA or specific early zygotic mRNAs. These results are consistent with the findings of previous studies demonstrating the importance of miRNAs in differentiation and development [4,14].

MiR-23, which exhibited pattern A (Fig. 3A), is known to target important genes in neuronal development [15]. It is present in adult fish tissues, frog ovaries [16] and mammalian neuronal cells [17]. Mir-23 is also known to down-regulate cell growth when inhibited in cell lines [18]. In rainbow trout embryos, omy-miR-23 is abundantly expressed in unfertilized eggs but is down regulated after fertilization. With limited understanding of the targets of this miRNA, the importance of this miRNA in early embryonic development is not clear.

Transcript level of omy-miR-26, member of pattern B, increased after onset of EGA (Fig. 3B). MiR-26 is induced under hypoxic conditions and reduces pro-apoptotic signals [19]. Expression of miR-26 gains relevance because fish eggs undergo embryonic development in aquatic environments where dissolved oxygen is often in short supply. Mir-26 also produces caspase inhibitory effects [20] which could have long ranging consequences in early metabolism of stored glycoproteins in trout embryos.

Among the miRNAs of pattern C that showed a particular increase after EGA at 5-dpf is omy-miR-21. omy-miR-21 was the most abundant miRNA in our library. Similarly, miR-21 was the most abundant miRNA accounting for ~40% of all cloned miRNAs in zebrafish [14]. This high abundance of miR-21 indicates an important role in fish embryonic development. The expression of miR-21 increases in zebrafish embryos at 1-dpf [14]. MiR-21 is a ubiquitous miRNAs being implicated as an anti-apoptotic factor [21,22] and an oncogene [23]. Suppression of miR-21 causes down-regulation of apoptosis related proteins like bcl2 leading to increase in apoptosis [22]. However in HeLa cells, inhibition of miR-21 causes profound increase in cell growth [18]. Rapidly dividing embryos mimic dividing cancer cells, but in our embryonic samples, miR-21 is up-regulated at a particular stage, 5-dpf, after active large scale division begins (Fig. 3C). It is possible that expression of miR-21 is essential to coordinate the cell division/cell growth in early embryos.

Similar to miR-21, miR-92 showed an increased expression at 5-dpf stage (Fig 3C). This miRNA was previously shown to be ubiquitously expressed in adult tissues and embryonic stem cells, indicating that it controls cell functions common to embryonic and adult tissues [24]. Expression of miR-92 is ubiquitous in early medaka and zebrafish embryos and becomes restricted to highly proliferative tissues late in development [25]. Polycystronic expression of miR-17-92 is associated with some types of cancers [26,27]. Transgenic over-expression of this cluster promotes cell proliferation and prevents differentiation in lung epithelial progenitor cells [28]. In rainbow trout, expression of miR-92 is abundant in embryos at 5-dpf. We suggest that miR-92 is involved in making an embryo competent of proliferation and it may also play a role in keeping these early cells un-differentiated.

### Expression of Stat3 and Dicer during embryonic development

Recently, it was shown that activation of miRNA-21 is necessary for the survival of myeloma cells [29]. This induction is mediated by signal transducer and activator of transcription 3 (Stat3). Stat3 proteins activate transcription and become functional with tyrosine phosphorylation by various cytokines [30,31]. Expression of miR-21 is indirectly but strictly dependent on Stat3 [29]. Using real time PCR, we measured the expression of Stat3 in rainbow trout embryos. As shown in Fig. 3, Stat3 transcript level is low in unfertilized eggs. It increases gradually, reaching a peak in 1-dpf embryos, and declines afterwards (Fig. 3E). The abundance of Stat3 transcripts does not appear to correlate with the level of omy-miR-21 which shows a peak accumulation in 5-dpf embryos. It is possible that Stat3 is involved in activation of other miRNAs in addition to miR-21 and thereby regulating degradation of maternal mRNAs in early embryos.

Dicer is a RNase III enzyme with two catalytic subunits [32,33] involved in processing of all miRNAs [33,34]. If miRNAs are involved in maternal mRNA degradation, Dicer must be present during EGA transition. Therefore, we determined the expression of Dicer gene in early stage embryos. We found that Dicer transcripts are detectable throughout the embryonic stages analyzed with elevated expression in embryos at 2-dpf (Fig. 3F). Embryonic genome activation occurs at mid-blastula stage in fish. According to Bobe and coworkers (2000), mid-blastula stage of rainbow trout embryos (cultured at ~12°C) occurs 2-dpf [35,36]. So, maternal mRNA degradation and EGA in rainbow trout could occur around 2-dpf. Peak expression of Dicer at 2-dpf, the time of maternal mRNA degradation and initiation of EGA, could indicate its involvement in miRNA processing during that period. As shown in Fig. 3, many miRNAs, after onset of EGA, start to increase their expression, indicating that they are being processed by Dicer around 2-dpf when Dicer transcript is highly abundant.

### Tissue distribution profiles of miRNAs in adult fish

**Figure 4:**
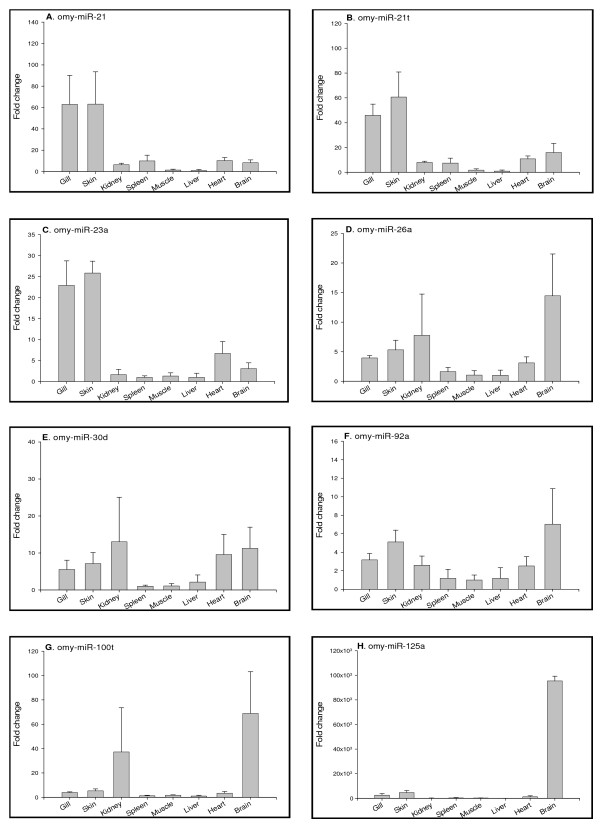
**Tissue distributions of miRNAs (A to H) analyzed by real-time PCR.** Quantity of each miRNA was normalized to Histone H2A. The means of the normalized gene expression values for each tissue were calculated and expressed as relative fold changes (n = 3, mean ± SEM).

**Figure 5:**
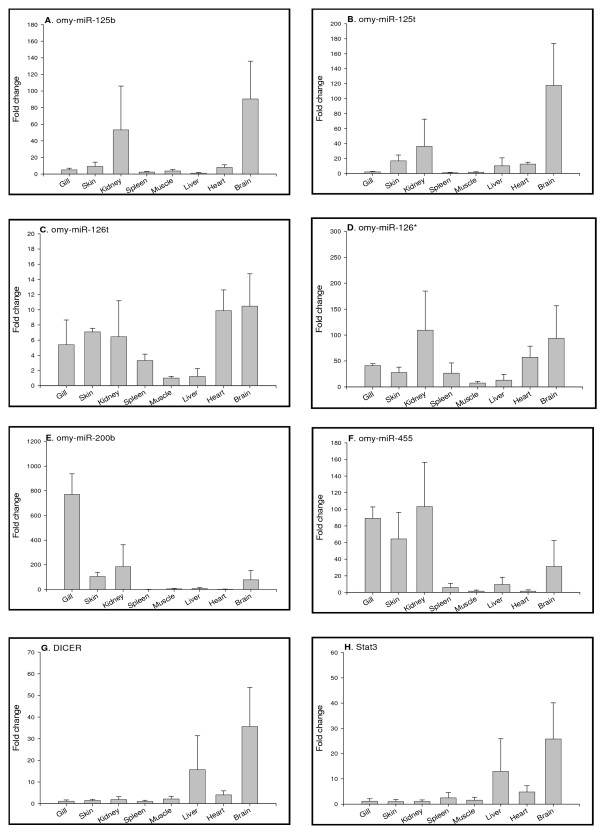
**Tissue distributions of miRNAs (A to F), Dicer (G) and Stat3 (H) transcripts analyzed by real-time PCR. **Quantity of each miRNA or mRNA was normalized to Histone H2A. The means of the normalized gene expression values for each tissue were calculated and expressed as relative fold changes (n = 3, mean ± SEM).

Quantifying miRNAs in different tissues is an important initial step to investigate functions of miRNAs. Tissue distribution of miRNAs provides essential baseline references to analyze variation of miRNA expression under various physiological conditions. To analyze the tissue distribution of the newly identified rainbow trout miRNAs, we performed real time PCR to determine the expression levels of all 14 miRNAs in 8 rainbow trout tissues that include gills, skin, kidney, spleen, muscle, liver, heart and brain. Tissue distribution profiles of these miRNAs are shown in Fig 4 and Fig 5. Some miRNAs, such as omy-miR26a omy-miR30d, omy-miR92a, omy-miR126t and omy-miR126*, demonstrated ubiquitous expression in all tissues examined. The ubiquitous nature of these miRNAs expression suggests that they might be associated with fundamental functions, such as metabolism [37]. On the other hand, we identified miRNAs with highly differential patterns of tissue distribution. For example, omy-miR125a shows prominent expression in brain, and miRNAs like omy-miR21, omy-miR21t, omy-miR23a and omy-miR455 are mainly expressed in the epithelial tissues (gills and skin). Other miRNAs, such as omy-miR100t, omy-miR125b and omy-miR125t, are highly abundant in kidney and brain. Tissue-specific expression of these miRNAs is indicative of tissue-specific functional roles of these miRNAs in regulating gene expression. Fig. 5 also shows the expression of Dicer (Fig. 5G) and Stat3 (Fig. 5H) in various tissues. Both gene transcripts show similar tissue expression patterns, being abundant in brain and liver.

Conservation of the tissue expression of individual miRNAs across different species suggests their fundamental physiological roles. Tissue expression patterns of some miRNAs are conserved between rainbow trout and other species. For example, the ubiquitous expression of miR-26a and miR-92a and prominent brain expression of miR-125a in rainbow trout was also observed in mammals and zebrafish, and miR-23a, mainly expressed in the rainbow trout epithelial tissues (gills and skin) is also highly expressed in mammalian and zebrafish epithelial tissues such as lung (mammals) and fins (zebrafish) [8]. On the other hand, miR126* that has restricted expression in mammals and zebrafish [8] appears to be present ubiquitously in rainbow trout tissues, suggesting that certain miRNAs may have species-specific functions.

## Conclusion

In this study we report the first cloning and expression of miRNAs from rainbow trout. Four novel miRNAs for rainbow trout were identified, emphasizing species-specific distribution and functions of miRNAs. Distinct patterns of expression for the cloned miRNA during early embryogenesis were observed and some miRNAs showed characteristic up-regulated expression during EGA. Both Dicer and Stat3 are abundantly expressed in rainbow trout embryos around the time of EGA, indicating the importance of these proteins in early embryonic development. We propose that activation of Stat3 leads to the activation of miRNA-21 and possibly other miRNAs. These miRNAs play an important role in degrading maternally inherited mRNAs, an essential step for normal embryonic development.

## Methods

### Collection of samples

Animals were reared under standard conditions. Rainbow trout eggs were fertilized and incubated at ~13°C in a flow through system in dark at the National Center for Cool and Cold Water Aquaculture (Kearneysville, WV). Unfertilized eggs (0 days post-fertilization) and developing embryos were collected at 1, 2, 3, 5 and 6 days post fertilization (dpf). Various somatic tissues including gill, skin, kidney, spleen, muscle, liver, heart and brain were also collected from adult fish. All samples were frozen in liquid nitrogen and stored at -80°C until total RNA isolation.

### MicroRNA cloning

Total RNA from unfertilized eggs and early embryos (1–6 dpf) was isolated using TRIzol™ reagent (Invitrogen, Carlsbad, CA) and pooled for miRNA cloning using the miRCat™ small RNA cloning kit (IDT DNA, Coralville, IA) following manufacturer's instructions with minor modifications. In brief, 500 μg of pooled total RNA was size fractionated using 12% denaturing polyacrylamide gel electrophoresis (PAGE) as described by [38]. Excised gels were homogenized in water and heated for 15 minutes at 70°C to solubilize small RNA and 3' linkers (5' phosphorylated) were ligated to the small RNA fraction in the absence of ATP. This was again size fractionated using 12% PAGE and 5' linkers were ligated in the presence of ATP. This mixture was then reverse transcribed using primer complementary to the 3' linker sequence and PCR amplified using primers on both linkers. Amplified products were *BanI *digested, concatemerized and cloned using TOPO TA cloning kit (Invitrogen, Carlsbad, CA). Transformed bacterial cells were plated and grown overnight. Individual colonies were picked and screened for presence of inserts by colony PCR. Clones with inserts were sequenced and sequence data were analyzed by BLAST search against the Sanger miRNA database [39]. MicroRNAs were identified and named based on sequence homology to published miRNAs according to the universal nomenclature [11].

### Quantitative real-time PCR for miRNAs, Dicer and Stat3

The expression of miRNAs, Dicer and Stat3 was measured using quantitative real-time PCR. Total RNA from different stage embryos (n = 3 pools, 5 embryos/pool) and adult tissues (n = 3) was isolated using TRIzol™ reagent (Invitrogen) followed by DNAse treatment. One μg of DNAse-treated RNA was converted to cDNA using miScript reverse transcriptase mix (Qiagen, Valencia, CA), a blend of enzymes comprising poly(A)polymerase and reverse transcriptase, and a mixture of oligo-dT (containing a universal tag sequence) and random primers. The cDNA was then used for real time PCR quantification of miRNAs (using a miRNA target-specific primer and the miScript Universal Primer (Qiagen) and Dicer or Stat3 mRNA (using gene-specific primers).

Real-time PCR miRNA target-specific primers are showed in Table 1. Primers for rainbow trout Dicer and Stat3 were designed based on their corresponding cDNA sequences (AY523839 for Dicer and U60333 for Stat3) in the NCBI database [40]. Primers for Dicer are: forward, AGGAGGCAGTGCTACCCTAAA; reverse, AAGTTGAGTTCGTCAGGCAGA. Primers for Stat3 are: forward, GCTGGACAACATCATTGACCT; reverse, GTGACTGCCTCCCTCCTTACT). The rainbow trout Histone H2A gene (TC85036 in TIGR database) was used as endogenous control. Primers for this gene are: forward, TCCCCAAGAAGACTGAGAAGG; reverse, TTTGTTGAGCTAGGTGGTTGG. All primers were designed using Primer3 software [41]. Quantitative real time PCR was performed in duplicate for each cDNA sample on a Bio-Rad iCycler iQ Real-Time PCR Detection System using iQ™ SYBR^® ^Green Supermix (Bio-Rad, Hercules, CA) in 25-μl reaction volumes containing 300 nM of each primer and cDNA derived from 0.05 μg of total RNA. Standard curves for all miRNAs, Dicer, Stat3 and the endogenous control were constructed using 10 fold serial dilutions of a pooled cDNA sample. Standard curves were run on the same plate with the samples. Threshold lines were adjusted to intersect amplification lines in the linear portion of the amplification curve and cycles to threshold (Ct) were recorded. For each sample, the quantity of miRNA/target gene mRNA and the reference gene mRNA (Histone H2A) was determined from respective standard curves. The quantity of miRNA/target gene mRNA was then divided by the quantity of the reference gene to obtain a normalized value. Mean differences in expression levels were reported as relative fold changes using the lowest expression value as a calibrator. This was done by designating the tissue or embryonic stage with the lowest expression value as a calibrator and dividing the means of other samples by the mean of the calibrator (calibrator mean divided by itself equals one). Specificity of amplification of each transcript was confirmed by melting curve analysis using iCycler software (Bio-Rad, Hercules, CA).

### Statistical analysis

One-way analysis of variance (ANOVA) was performed on mean gene expression levels using SigmaStat (version 3.11) software (Aspire Software International, Leesburg, VA). When one-way ANOVA showed significant effects, multiple mean comparisons were made using the Holm-Sidak method. Differences between groups were reported as significant (denoted by a different alphabet in figures) if p < 0.05.

### Bioinformatics analysis

Sequences were analyzed for any homology to published miRNAs by BLAST searching of the Sanger miRNA database [39]. Multiple sequence alignments were carried out using BioEdit software (Ibis Biosciences, Carlsbad, CA).

## Authors' contributions

RR was responsible for miRNA cloning and drafted the manuscript. MS generated the embryonic and tissue gene expression data and reviewed the manuscript. SG provided embryonic samples. CR participated in coordination. JY was responsible for project development and is the corresponding author. All contributing authors reviewed and approved the final copy of this manuscript.
